# Anti-inflammatory and antioxidant effects of muscarinic acetylcholine receptor (mAChR) activation in the rat hippocampus

**DOI:** 10.1038/s41598-019-50708-w

**Published:** 2019-10-02

**Authors:** Monica Frinchi, Domenico Nuzzo, Pietro Scaduto, Marta Di Carlo, Maria F. Massenti, Natale Belluardo, Giuseppa Mudò

**Affiliations:** 1 Department of Biomedicine, Neurosciences and Advanced Diagnostic, div. of Human Physiology, University of Palermo, 90134 Palermo, Italy; 2 Institute of Biomedicine and Molecular Immunology “Alberto Monroy” (IBIM), Consiglio Nazionale delle Ricerche (CNR), 90146 Palermo, Italy; 3 Department of Sciences for Health Promotion and Mother and Child Care “Giuseppe D’Alessandro”, University of Palermo, 90134 Palermo, Italy

**Keywords:** Alzheimer's disease, Neuroimmunology

## Abstract

Recently we found that acute treatment with Oxotremorine (Oxo), a non-selective mAChRs agonist, up-regulates heat shock proteins and activates their transcription factor heat shock factor 1 in the rat hippocampus. Here we aimed to investigate: a) if acute treatment with Oxo may regulate pro-inflammatory or anti-inflammatory cytokines and oxidative stress in the rat hippocampus; b) if chronic restraint stress (CRS) induces inflammatory or oxidative alterations in the hippocampus and whether such alterations may be affected by chronic treatment with Oxo. In the acute experiment, rats were injected with single dose of Oxo (0.4 mg/kg) and sacrificed at 24 h, 48 h and 72 h. In the CRS experiment, the rats were exposed for 21 days to the CRS and then were treated with Oxo (0.2 mg/kg) for further 10 days. The acute Oxo treatment showed an ability to significantly reduce reactive oxygen species (ROS), singlet oxygen (^1^O_2_), pro-inflammatory cytokines levels (IL-1β and IL-6) and phosphorylated NF-κB-p65. Acute Oxo treatment also increased superoxide dismutase (SOD)-2 protein levels and stimulated SOD activity. No differences were detected in the anti-inflammatory cytokine levels, including IL-10 and TGF-β1. In the group of rats exposed to the CRS were found increased hippocampal IL-1β and IL-6 levels, together with a reduction of SOD activity level. These changes produced by CRS were counteracted by chronic Oxo treatment. In contrast, the upregulation of ROS and ^1^O_2_ levels in the CRS group was not counteracted by chronic Oxo treatment. The results revealed a hippocampal anti-inflammatory and antioxidant effect of Oxo treatment in both basal conditions and anti-inflammatory in the CRS rat model.

## Introduction

Several reports have showed that cholinergic system in the brain may modulate several function, including neurogenesis, neuroprotection, neuronal differentiation and plasticity^1–5^. The family of muscarinic acetylcholine receptors (mAChRs) is composed of five subtypes (M_1_R-M_5_R), with M_1_R and M_3_R highly expressed in the hippocampus, where they regulate neuronal plasticity, neuroprotection, cognitive functions and behaviors^1,6,7^. Recently, we found that the non-selective mAChRs agonist Oxotremorine (Oxo) may transactivate the fibroblast growth factor receptor (FGFR) and increase neurite outgrowth in the hippocampal primary neuronal cultures^8^. More recently, we evidenced that Oxo treatment recovered rats from the stress-induced anxiety-like behavior, and restored fibroblast growth factor 2 (FGF2) and brain derived neurotrophic factor (BDNF) levels in ventral hippocampus and prefrontal cortex^7^, two brain regions considered responsible of chronic restraint stress (CRS) induced anxiety. In another work, we found that Oxo up-regulates heat shock proteins and activates their transcription factor heat shock factor 1 (HSF1) in the rat hippocampus^9^. Heat shock proteins, in addition to their importance in protecting neurons from protein mis-folding and aggregation, are also regulators of inflammation and oxidative stress^10^.

In the present work we aimed to investigate if mAChR may regulate in the rat hippocampus pro-inflammatory or anti-inflammatory cytokines and responses to oxidative stress. Concerning the involvement of mAChR activation in the inflammatory response, only two papers are available and both reported scopolamine-mediated increase of tumor necrosis factor-α (TNF-α) and stimulation of transcription factor nuclear factor-kB-p65 (NF-κB-p65) pathway in the rat hippocampus^11^ and in immortalized murine microglia BV-2 cells^12^. Since both investigations have used scopolamine, it is not clear if cholinergic neurotransmission activation is responsible for anti-inflammatory effects or if other neurotransmitters are involved as consequence of mAChRs inhibition. For example Young Jin Jang *et al*.^11^ reported that decaffeinated coffee counteracted the hippocampal increase of TNF-α and phosphorylation of inhibitor protein-κBα and NF-κB-p65 induced by scopolamine treatment. Similarly, Jung *et al*.^12^ reported that mangiferin, a compound with pleiotropic function including inhibition of Acetylcholinesterase, may counteract the effects of scopolamine. Concerning the role of mAChRs in neuronal response to oxidative stress, there are few discordant data. Indeed, some data reported that mAChR activation increases neuronal vulnerability to oxidative stress^13^, while others outlined that mAChR stimulation can induce reactive oxygen species (ROS), which may be involved, as second messengers, in cell-signaling pathways^14,15^.

Recent studies have developed lots of data concerning the stress-induced anxiety-depression and generation of hippocampal inflammation and oxidative stress. In this context, several reports have shown increased levels of cytokines, such as interleukin-1β (IL-1β) and interleukin-6 (IL-6) in experimental models of anxiety-depression^16^. Indeed, prolonged restrain stress causes increase of IL-6^17^, and chronic stress^18^ or social stress^19^ may induce neuroinflammation by increasing NF-κB^20,21^ and IL-1β in the brain^22^. Interestingly, knockout mice for IL-6 gene expression showed protection against stress-induced anxiety-depression-like behaviors^23^. In addition to inflammation, oxidative stress has also been showed to take part in the anxiety-depression and other psychiatric disorders^24–26^. During chronic stress, overproduction of ROS induces oxidative process dysregulation, producing neuronal damage^27^. The protection against oxidative stress needs antioxidant enzymes such as superoxide dismutase (SOD), which catalyze the dismutation of superoxide anion (O_2_^−^) to oxygen and hydrogen peroxide (H_2_O_2_). In this context, Möller *et al*.^28^ reported that post-weaning social isolation induces SOD activity in the brain, whereas the treatment with antipsychotic clozapine counteracts both the behavioral alterations and changes of redox activity.

According to the hypothesis of inflammatory and oxidative damage involvement in stress- induced anxiety-depression, the antidepressant effect of several drugs has been linked to a reduction of pro-inflammatory cytokines in the rat hippocampus^29^. In this context, antidepressant effect of ketamine has been attributed to both inhibition of pro-inflammatory responses^30,31^ and the upregulation in hippocampal levels of IL-6 and IL-1β, probably in relation to different treatment protocols^32^. Treatment with agonist of β-adrenergic receptors in stressed animals increases the expression of IL-1β in the brain^33,34^, whereas β-adrenergic receptor antagonist counteracts anxiety and activation of microglia produced by repeated social defeat^35^. Moreover, the antidepressant fluoxetine may reduce the levels of pro-inflammatory cytokines, including IL-1β^36^. Based on published data and our findings reporting the effects of Oxo treatment in reducing the anxiety-like behavior induced by CRS^7^, in the present work we aimed to examine whether our model of CRS-induced anxiety-like behavior shows pro-inflammatory or oxidative alterations in the hippocampus and whether such alterations may be affected by treatment with Oxo. In order to verify the involvement of mAChR receptors activation in pro-inflammatory or anti-inflammatory cytokines regulation we have chosen to accurately investigate, by time-course analysis, the effects of acute treatment with Oxo, a potent non-selective mAChR agonist, on cytokines levels. For this purpose, we have analyzed two canonic pro-inflammatory cytokines, IL-1β and IL-6, and two anti-inflammatory cytokines, interleukin-10 (IL-10) and transforming growth factor β1 (TGF-β1). In addition, we have also analyzed the effects of Oxo on the NF-κB, ROS levels, cytosolic SOD (SOD1) and mitochondrial SOD (SOD2) both as protein and activity levels. In addition, as mentioned above, we examined whether inflammatory or oxidative alterations were present in the hippocampus of rats exposed to CRS and whether such alterations were affected by chronic treatment with Oxo.

## Results

### Cortisol levels

Among the glucocorticoid regulating the stress responses in rodents, we have analyzed cortisol levels instead of corticosterone levels, widely used in literature, according with previous studies showing similarity between cortisol and corticosterone levels^37^.

#### Cortisol levels in rats after acute Oxo treatment

**Figure 1:**
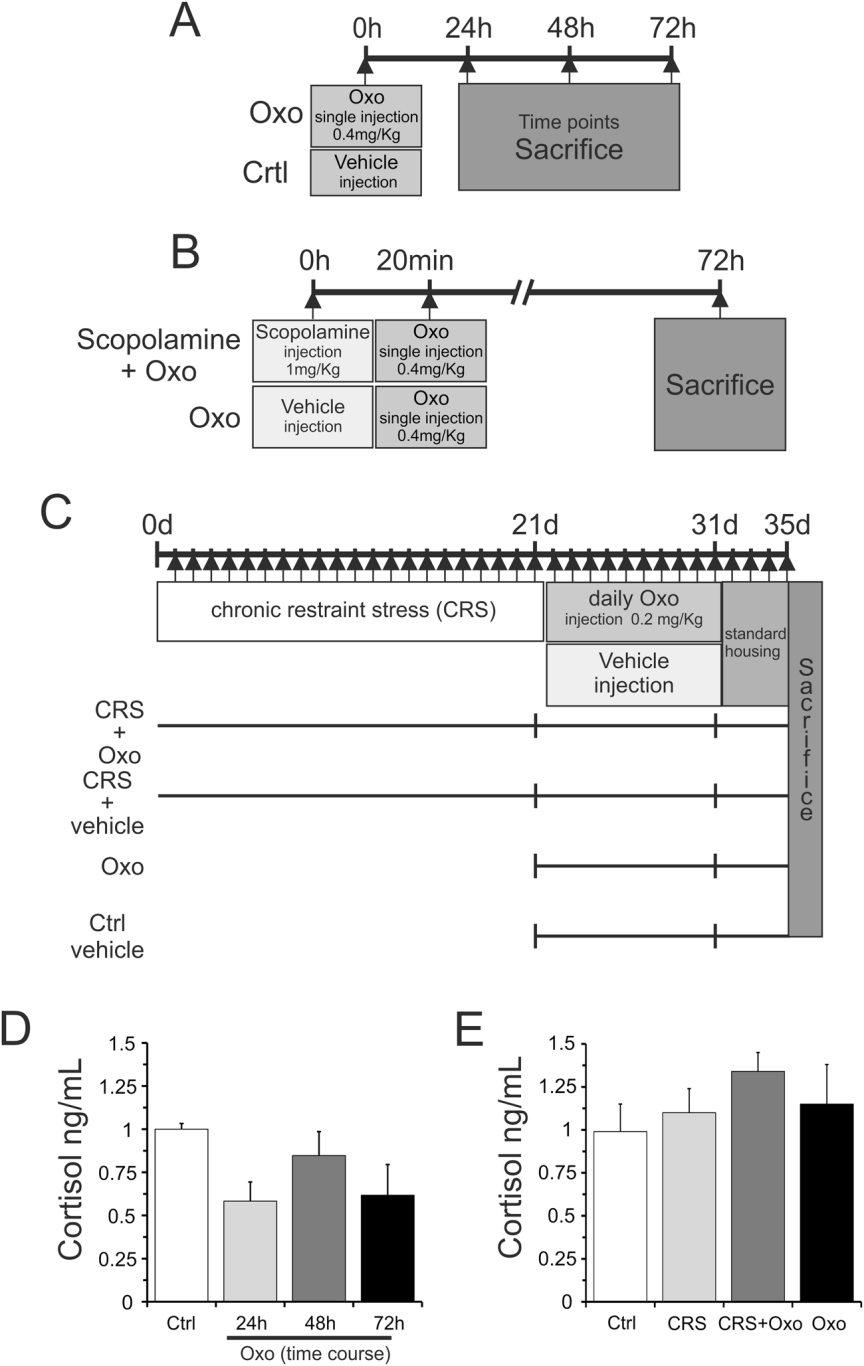
Schedule of treatment experimental procedures and serum cortisol levels. (**A**) Experimental schedule of time-course treatment with single injection of Oxo (0.4 mg/kg) or saline (Ctrl, vehicle) and sacrifice after 24 h, 48 h and 72 h. (**B**) Pretreatment with 1 mg/kg of scopolamine 20 min before single injection of Oxo treatment (0.4 mg/kg) or single injection of Oxo and sacrifice after 72 h. (**C**) Experimental schedule of chronic restraint stress (CRS) for 21 days, chronic treatment with Oxo (0.2 mg/kg) for 10 days and sacrifice after 4 days. (**D**) Quantitative analysis of serum cortisol levels after single injection of Oxo (0.4 mg/kg) and sacrifice at different time points (24 h, 48 h and 72 h). (**E**) Quantitative analysis of serum cortisol levels after CRS and chronic treatment with Oxo (0.2 mg/kg) for 10 days. Data in (**D**,**E**) represent Mean ± S.E.M.

The results of the cortisol levels after acute Oxo treatment (0.4 mg/kg) are reported in Fig. 1D. The analysis of cortisol levels showed a trend of reduction in all the examined time-points, but in none of them it becomes significant as compared to control group.

#### Cortisol levels in rats exposed to CRS and their regulation by chronic Oxo treatment

The results of cortisol levels in rats exposed to CRS and related effects of chronic Oxo treatment are reported in Fig. 1E. The analysis of cortisol levels showed a trend of increase in all experimental groups, but no significance was found for stress effect (F_(1,28)_ = 0.827, p > 0.05), nor for Oxo treatment (F_(1,28)_ = 1.48, p > 0.05) and interaction (stress × treatment: F_(1,28)_ = 0.057, p = 0.81).

### Oxo effect on pro-inflammatory and anti-inflammatory cytokine levels in the rat hippocampus

**Figure 2:**
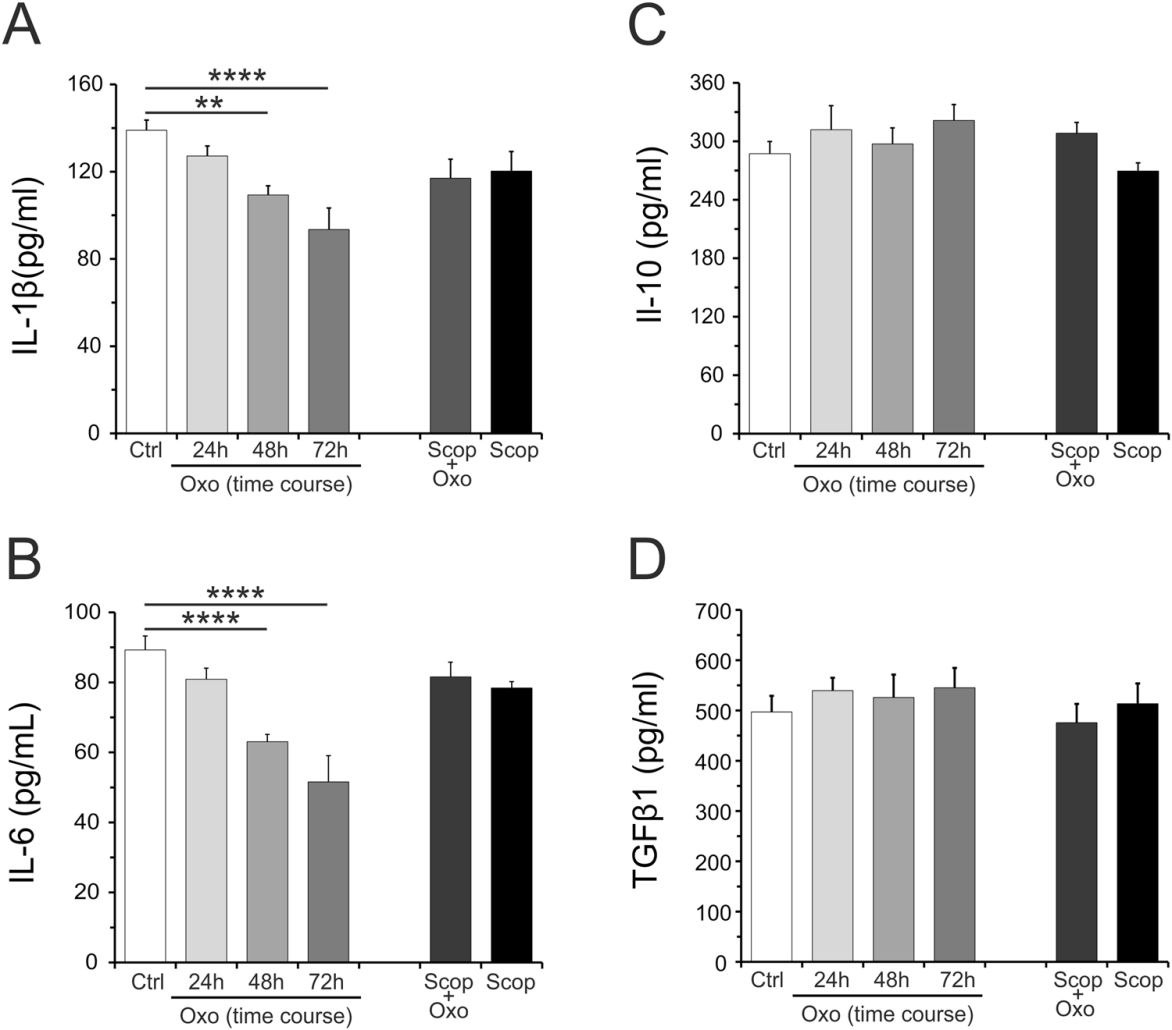
Oxo effect on pro-and anti-inflammatory cytokines protein levels in the hippocampus. ELISA analysis of IL-1β (**A**) and IL-6 (**B**) protein levels at different time-points (24 h, 48 h and 72 h) after acute Oxo treatment revealed a significant decrease at 48 h and 72 h as compared to control group (Ctrl). Scopolamine (Scop), injected 20 min before acute Oxo treatment, completely blocked Oxo effect. Scopolamine alone did not show significant changes of both IL-1β and IL-6. The *ELISA* analysis of IL-10 (**C**) and TGF-β1 (**D**) protein levels did not show significant variation in all experimental groups. Data are reported as means ± SE. PLSD: **P < 0.01, ****P < 0.0001.

In order to study the effects of Oxo treatment on hippocampal pro-inflammatory and anti-inflammatory cytokines levels, we performed an acute treatment with Oxo at dose of 0.4 mg/kg and rats were killed at three different time points (24 h, 48 h and 72 h) after Oxo treatment. The experimental design is schematized in Fig. 1A. The levels of pro-inflammatory cytokines, IL-1β and IL-6, measured by ELISA kit, were found significantly reduced at 48 h and 72 h after acute Oxo treatment (Fig. 2A,B). The levels of anti-inflammatory cytokines, including IL-10 and TGF-β1, were not affected by acute Oxo treatment in any examined time-points (Fig. 2C,D).

**Figure 3:**
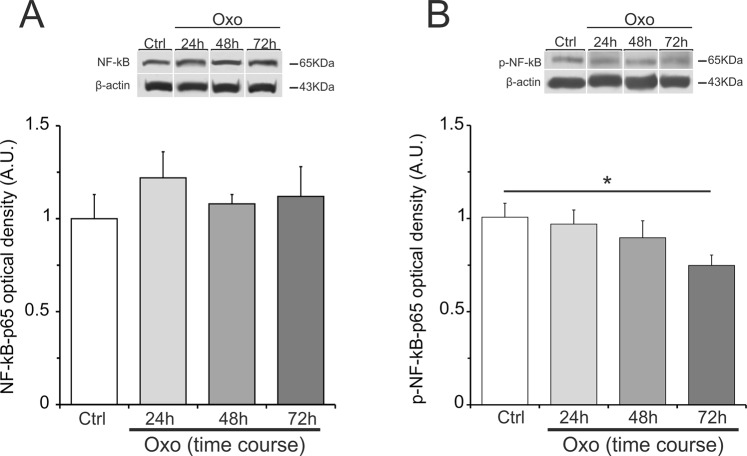
Oxo effect on NF-κB-p65 and pNF-κB-p65 protein levels in the hippocampus. Cropped band-images of representative Western blot results of NF-κB-p65 and pNF-κB-p65 protein levels at different time-points (24, 48 and 72 h) after acute Oxo treatment. (**A**) NF-κB-p65 protein levels did not show changes following Oxo treatment as compared to control (Ctrl). (**B**) pNF-κB-p65 protein levels showed a significant decrease at 72 h after acute Oxo treatment as compared to control group. Full-length band-lines are presented in Supplementary Fig. S1. Data are reported as means ± SE. PLSD: *P < 0.05.

Next, we examined the transcription factor NF-κB, which is extensively studied for its role in regulating expression of IL-1β and IL-6. The NF-κB-p65 subunit protein levels were found unchanged (Fig. 3A), whereas phosphorylation of NF-κB-p65 (pNF-κB-p65) resulted significantly decreased at 72 h after acute Oxo treatment (Fig. 3B).

### Oxo effect on ROS levels, SOD1 and SOD2 proteins and activity levels in the rat hippocampus

**Figure 4:**
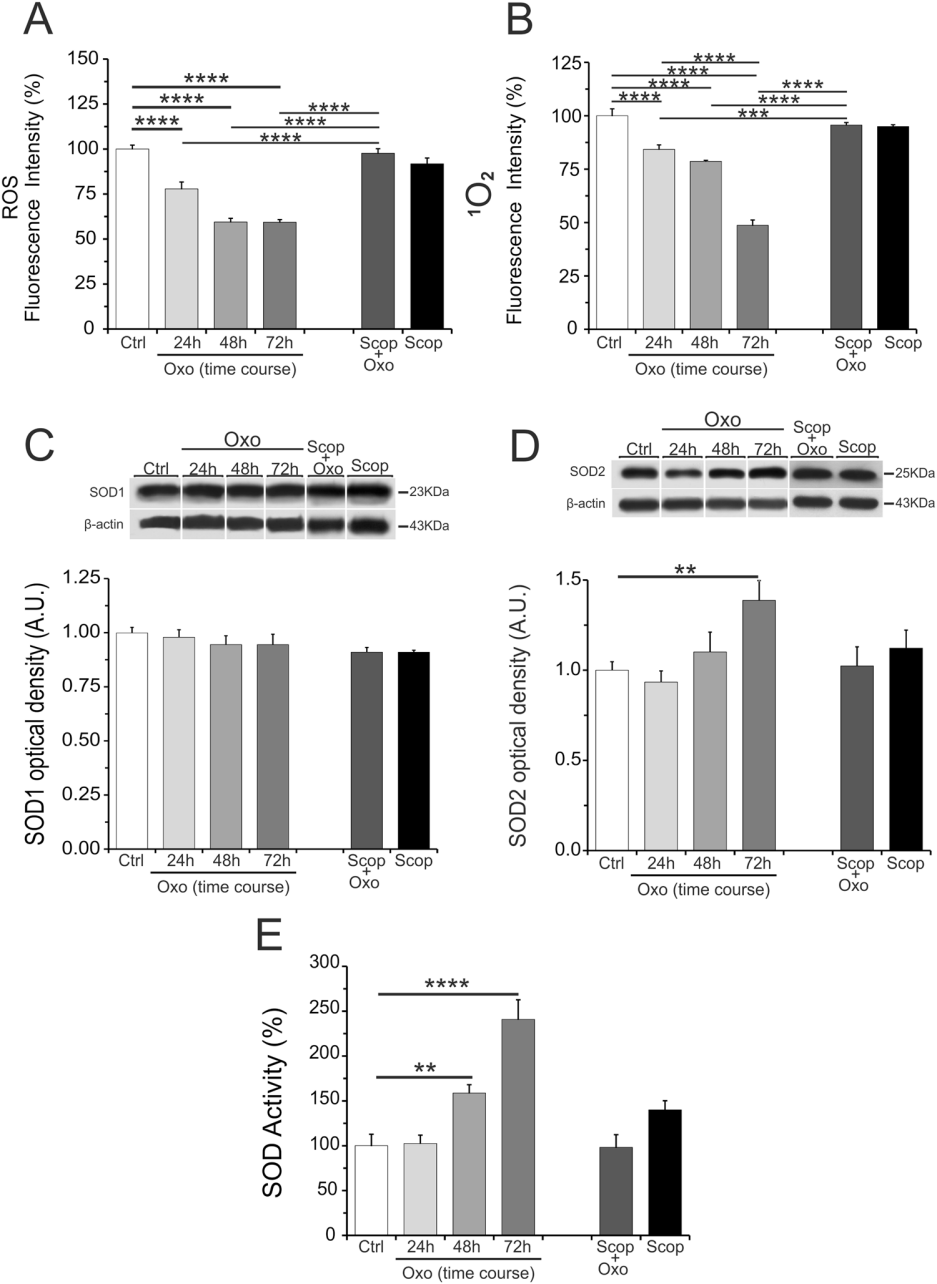
Oxo effect on ROS levels and on SOD1 and SOD2 protein levels and activity in the hippocampus. Cropped band-images of representative Western blot results. (**A**) The analysis of global ROS levels following acute Oxo treatment revealed a significant decrease in all the studied time points (24 h, 48 h and 72 h) as compared to control group (Ctrl). (**B**) The selective analysis of ^1^O_2_ species levels following acute Oxo treatment revealed a significant decrease in all the studied time points (24 h, 48 h and 72 h) as compared to control group (Ctrl). The scopolamine (Scop), injected 20 min before of acute Oxo treatment, completely blocked Oxo effect on global ROS and ^1^O_2_ species levels. Scopolamine alone did not show significant changes of both global ROS and ^1^O_2_ species levels. (**C**,**D**) The analysis of SOD1and SOD2 protein levels at different time-points (24 h, 48 h and 72 h) revealed a significant increase only for SOD2 at 72 h as compared to control group. Scopolamine pretreatment completely blocked the increase of SOD2 protein levels. The group treated with scopolamine alone did not show significant change of SOD1 and SOD2 protein levels. (**E**) The analysis of SOD activity evidenced a significant increase of SOD activity at 48 h and 72 h after acute Oxo treatment as compared to control group. Scopolamine pretreatment completely blocked Oxo effect on SOD activity. Scopolamine alone did not show significant changes of SOD activity as compared to control group. Full-length band-lines are presented in Supplementary Fig. S2. Data are reported as means ± SE. PLSD: **P < 0.01, ***P < 0.001, ****P < 0.0001.

In this study, the ability of Oxo to affect oxidative stress in the hippocampus was evaluated by detecting ROS levels and SOD activity. Global ROS levels, detected using DCFH-DA assay, showed a significant reduction at all the studied time points following acute Oxo treatment (Fig. 4A). Among ROS, the selective evaluation of ^1^O_2_ species levels, detected using SOSG assay, also showed a significant reduction at all the studied time points after acute Oxo treatment (Fig. 4B). Therefore, the selective analysis of ^1^O_2_ species levels showed a similar pattern of global ROS levels. Next, we examined SOD1 and SOD2 protein levels and we found a significant increase of SOD2 at 72 h, whereas SOD1 was found unchanged at all the studied time points (Fig. 4C,D). The evaluation of SOD activity level revealed a significant increase at 48 h and 72 h after acute Oxo treatment (Fig. 4E). This increase of SOD activity level was in good correlation with the upregulation of SOD2 protein levels and with ROS reduction at 72 h after Oxo treatment.

### mAChR antagonist scopolamine pretreatment on Oxo effects

The specificity of the observed acute Oxo effects was verified using pretreatment with the non-selective mAChR antagonist scopolamine (1 mg/kg) administered 20 min before Oxo injection (0.4 mg/kg). The experimental design is schematized in Fig. 1B. For this study was used the time point of 72 h after Oxo treatment. The results showed that scopolamine completely blocked Oxo effects on IL-1β and IL-6 protein levels (Fig. 2A,B). The levels of IL-10 and TGF-β1, already not influenced by acute Oxo treatment, did not show any modifications following pretreatment with scopolamine (Fig. 2C,D). Similarly, scopolamine pretreatment completely blocked the ability of Oxo to reduce oxidative stress, by counteracting the increase of both SOD2 protein levels and SOD activity and restoring ROS and ^1^O_2_ levels (Fig. 4). The group treated with scopolamine alone (1 mg/kg) did not show significant changes in all the examined cytokines protein levels and in ROS or ^1^O_2_ production or SOD1 and SOD2 protein levels, including SOD activity (Figs 2 and 4).

### Cytokine levels in the hippocampus of rats exposed to CRS and their regulation by chronic Oxo treatment

**Figure 5:**
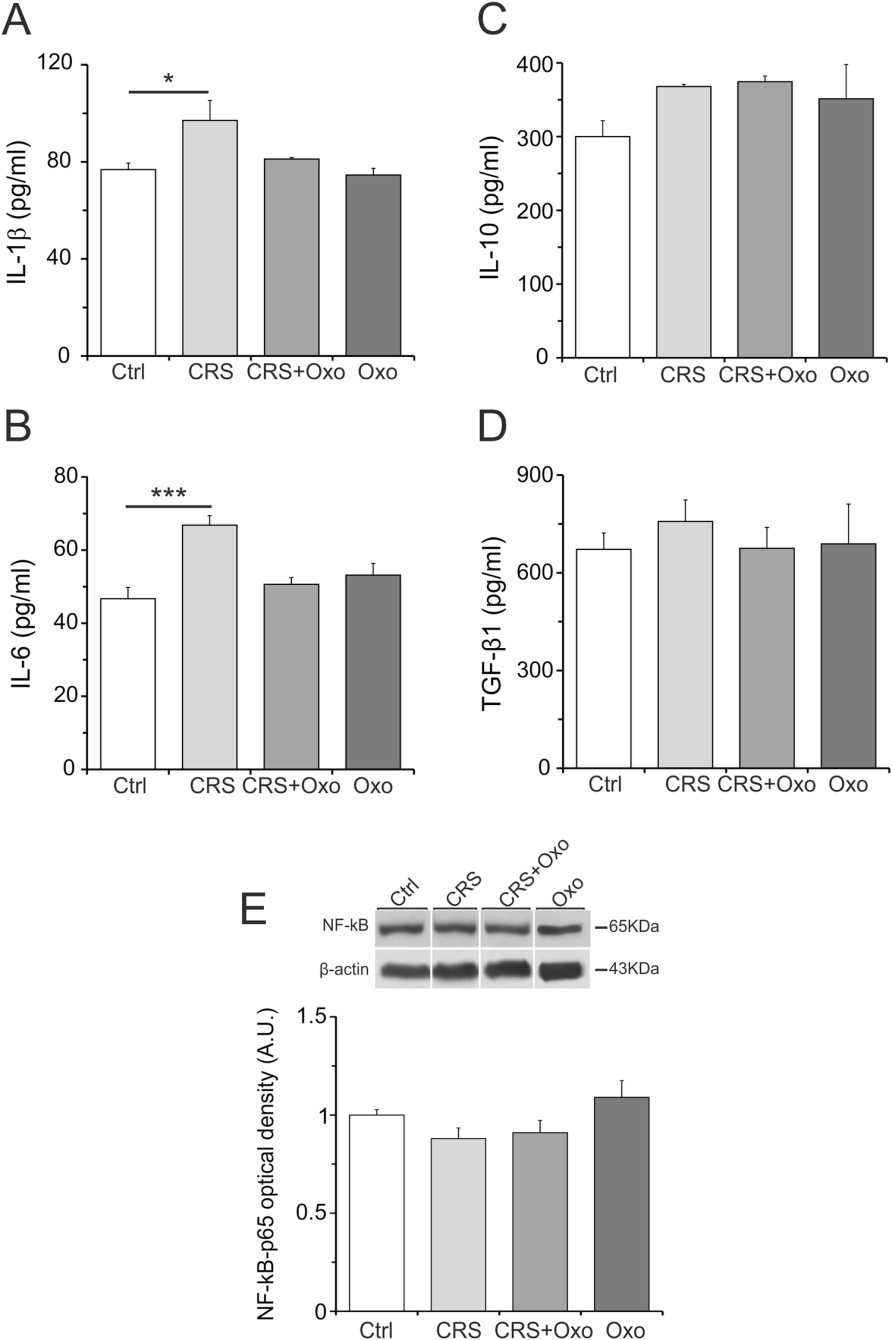
Pro-inflammatory and anti-inflammatory cytokine levels, including NF-κB-p65, in the hippocampus of rats exposed to CRS and their regulation by chronic Oxo treatment. ELISA analysis of IL-1β (**A**) and IL-6 (**B**) protein levels revealed a significant increase in CRS group. The chronic Oxo treatment completely blocked CRS effect on IL-1β and IL-6. Oxo treated group did not show significant changes of both IL-1β and IL-6. The ELISA analysis of IL-10 (**C**) and TGF-β1 (**D**) protein levels did not show significant variation in all experimental groups. (**E**) Cropped band-images of representative Western blot results. The analysis of NF-κB-p65 protein levels did not show significant changes in all experimental groups. Full-length band-lines are presented in Supplementary Fig. S3. Ctrl, Control group. Data are reported as means ± SE. t-test: *P < 0.05, ***P < 0.001.

Recently, as mentioned above, have been developed a lot of data concerning the stress-induced anxiety-depression and generation of hippocampal inflammation and oxidative stress. Accordingly, the effectiveness of some anxiolytic-antidepressant compounds, including Oxo, has been associated to the reduction of cytokines and oxidative stress in the hippocampus. Therefore, in the present work we examined whether CRS was characterized by hippocampal changes of inflammatory cytokines levels or oxidative stress and, consequently, if such alterations would be affected by chronic treatment with Oxo. The experimental design is schematized in Fig. 1C. The results showed that pro-inflammatory cytokines, IL-1β and IL-6, were significantly up-regulated in the CRS group (stress effect: IL-1β, F_(1,16)_ = 8.8, p < 0.02; IL-6, F_(1,16)_ = 10.4, p < 0.02), and that this increase was blocked by chronic Oxo treatment (treatment effect: IL-1β, F_(1,16)_ = 4.0, p = 0.8; IL-6, F_(1,16)_ = 3.2, p = 0.11) (stress × treatment effect: IL-1β, F_(1,16)_ = 2.26, p = 0.17; IL-6, F_(1,16)_ = 17.26, p < 0.01) (Fig. 5A,B). However, IL-1β and IL-6 levels in the group treated with chronic Oxo alone have not changed in comparison to control group, contrarily to reduction observed with acute Oxo treatment. The anti-inflammatory cytokines, TGF-β1 (treatment effect: F_(1,16)_ = 0.17, p = 0.69; stress effect: F_(1,16)_ = 0.2, p = 0.66; stress × treatment effect: F_(1,16)_ = 0.38, p = 0.55) and IL-10 (treatment effect: F_(1,16)_ = 1.22, p = 0.3; stress effect: F_(1,16)_ = 0.3, p = 0.12; stress × treatment effect: F_(1,16)_ = 0.73, p = 0.42), and the NF-κB-p65 protein levels did not show significant changes (treatment effect: F_(1,15)_ = 0.55, p = 0.47; stress effect: F_(1,15)_ = 4.1, p = 0.06; stress × treatment effect: F_(1,15)_ = 0.119, p = 0.66) in all experimental groups (Fig. 5C–E).

### Oxidative stress levels in the hippocampus of rats exposed to CRS and their regulation by chronic Oxo treatment

**Figure 6:**
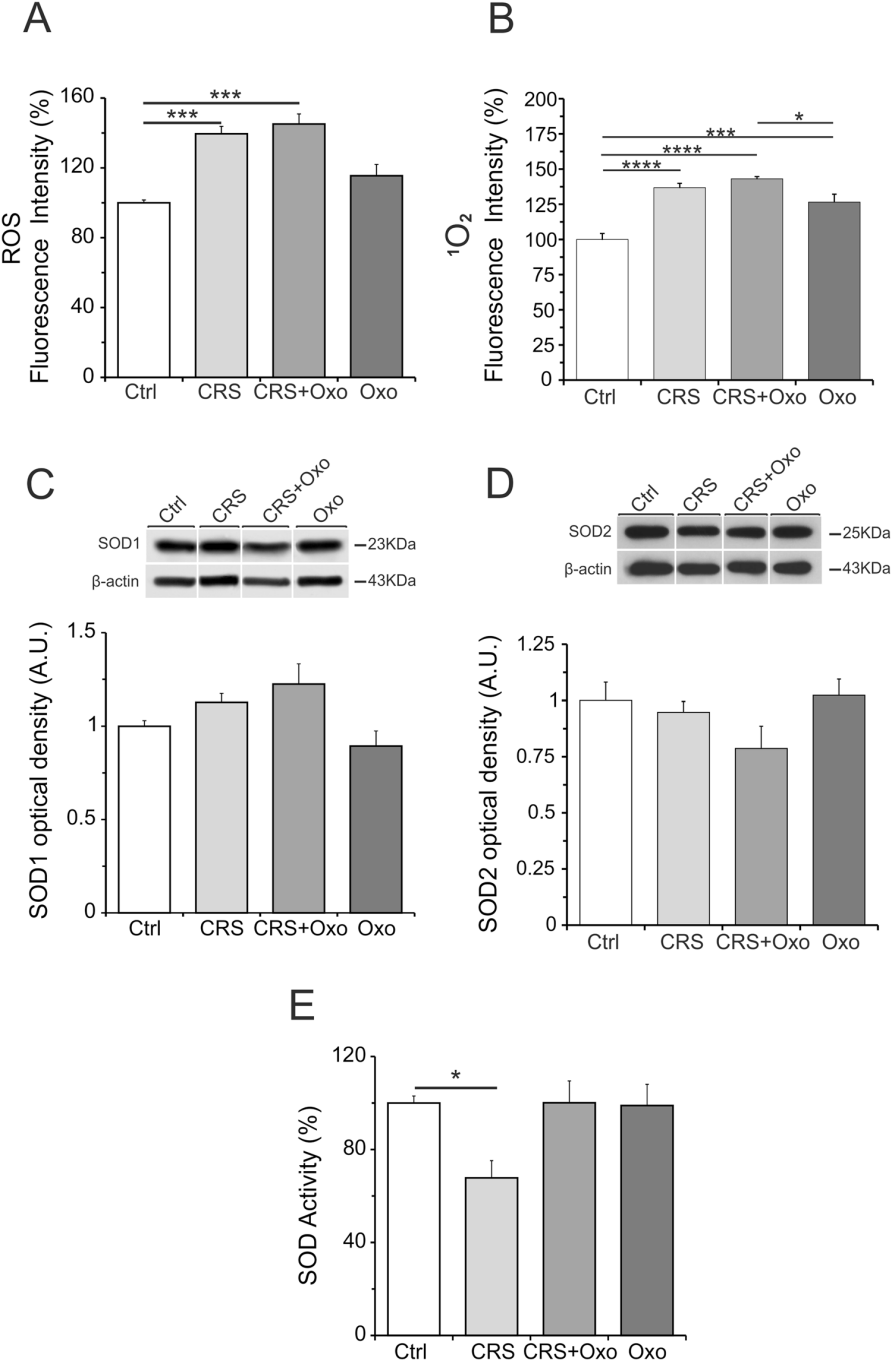
ROS levels, SOD1 and SOD2 protein levels and activity in the hippocampus of rats exposed to CRS and their regulation by chronic Oxo treatment. (**A**) The analysis of global ROS levels revealed increased levels of ROS in the CRS. Treatment of CRS group with chronic Oxo failed to counteract the ROS increase. (**B**) The selective analysis of ^1^O_2_ species levels revealed a significant increase in the CRS. Treatment of CRS group with chronic Oxo failed to counteract the ^1^O_2_ species increase. The group treated with Oxo alone showed a significant increase of ^1^O_2_ species levels as compared to control group. (**C**,**D**) The Western blotting analysis showed unchanged levels of SOD1 and SOD2 protein in all experimental groups. Cropped band-images are representative of Western blot results. Full-length band-lines are presented in Supplementary Fig. S4. (**E**) SOD activity level was found significantly reduced in the CRS group. Chronic Oxo treatment counteracted SOD activity reduction in the CRS group. Group treated with Oxo alone did not show any variations as compared to control group (Ctrl). Data are reported as means ± SE. t-test: *P < 0.05, ***P < 0.001, ****P < 0.0001.

According to experimental studies reporting the effects of oxidative stress in anxiety and depression pathogenesis^38^, here we detected a significant increase of global ROS levels in the CRS group (stress effect: F_(1,16)_ = 49.8, p = 0.0001) (Fig. 6A). However, chronic treatment of CRS group with Oxo failed to counteract this global ROS increase (treatment effect: F_(1,16)_ = 4.6, p = 0.64). In the group treated with chronic Oxo alone the global ROS levels although increased did not reach significance, revealing that chronic Oxo treatment did not show any inhibitory effects on ROS levels (stress × treatment effect: F_(1,16)_ = 1.02, p = 0.34) (Fig. 6A), contrarily to what was observed with acute Oxo treatment (Fig. 4A). The selective analysis of ^1^O_2_ species levels showed a similar pattern of global ROS levels, with the achievement of significant increase even in the group treated with Oxo alone (stress effect: F_(1,16)_ = 49.2, p < 0.0001; treatment effect: F_(1,16)_ = 18.7, p < 0.01; stress × treatment effect: F_(1,16)_ = 7.1, p = 0.024) (Fig. 6B).

SOD1 and SOD2 protein levels were found unchanged in all experimental groups (SOD1: stress effect: F_(1,16)_ = 4.44, p = 0.51; treatment effect: F_(1,16)_ = 0.029, p = 0.98; SOD2 = stress effect: F_(1,14)_ = 3.34, p = 0.095; treatment effect: F_(1,14)_ = 0.74, p = 0.4) (Fig. 6C,D), whereas SOD activity level was found significantly reduced in the CRS group (stress effect: F_(1,8)_ = 5.44, p < 0.048;) and that this decrease was counteract by chronic Oxo treatment (treatment effect: F_(1,8)_ = 5.0, p = 0.051) (treatment × stress effect: F_(1,8)_ = 6.27, p < 0.037) (Fig. 6E). Chronic Oxo treatment counteracted SOD activity reduction in the CRS group, whereas chronic Oxo treatment alone did not produce any changes (Fig. 6E), contrarily to increase observed with acute Oxo treatment (Fig. 4E).

## Discussion

Altogether, the present results indicate potential neuroprotective effects of acute Oxo due to its ability to reduce ROS and pro-inflammatory cytokines levels and to stimulate SOD activity. Interesting, these Oxo effects revealed a muscarinic modulation of ROS and pro-inflammatory molecules baseline levels in normal condition. This regulatory action toward pro-inflammatory factors seems to be specific, since Oxo treatment does not affect the anti-inflammatory IL-10 and TGF-β1. The results also highlight that chronic Oxo treatment may inhibit neuroinflammatory responses in the hippocampus of rats exposed to CRS.

As already indicated in the introduction section, only two papers are available in the context of anti-inflammatory Oxo effects and both reported the scopolamine-mediated elevation of TNF-α and the stimulation of NF-κB pathway in the rat hippocampus^11^ and in immortalized murine microglia BV-2 cell^12^. Therefore, the anti-inflammatory action observed in the present study by activation of mAChRs is consistent with the pro-inflammatory effects observed by previous studies with mAChRs antagonist scopolamine. However, in the present investigation we found that the treatment with mAChR antagonist scopolamine alone does not affect any of the studied cytokines.

In the hippocampus, IL-1β is released mainly by microglia but also by neurons and astrocytes^39^. Concerning IL-1β function, in addition to its pro-inflammatory role, low expression levels are involved in learning and memory, while chronically elevated IL-1β levels reduced memory and cognition^40,41^. Our observation of the inhibitory effect of mAChR activation on IL-1β baseline levels suggests a new mechanism through which the cholinergic system may regulate learning and memory.

Despite the fact that astrocytes and microglia are the main producers of IL-6 in the brain^42^, neuronal cells can also produce IL-6 in brain disease and injury or following prolonged neuronal activity^43^. Although several experimental conditions have shown up-regulation of IL-6 levels in glial cells and neurons^44^, no data are available on mAChR activation. IL-6 was found increased during neuroinflammatory response, such as brain infection and injury or in several brain diseases^44^. However, IL-6 was also found involved in the normal brain functions. For example inhibition of IL-6 expression produced improvement of long-term memory, suggesting a negative regulation of IL-6 in memory formation. As support to these results, mice with up-regulated IL-6 levels in the brain show impaired cognitive functions and deficits in learning^45^. In this study, the observed down-regulation of IL-6 in the hippocampus mediated by mAChR activation could then suggest new mechanism through which the cholinergic system may regulate learning and memory. However, from an anti-inflammatory point of view, this reduction of both IL-1β and IL-6 could explain the hypothesis, proposed by Borokivova^46^, according to which ACh levels decrease can favor neuroinflammatory events, as observed in Alzheimer disease.

On the contrary, TGF-β1 and IL-10, actively involved in anti-inflammatory responses^47,48^ were not regulated by mAChR activation, suggesting a mAChR selective regulation of pro-inflammatory cytokines rather than anti-inflammatory ones.

Neuronal cells possess substantial constitutive NF-κB activity, which can be triggered not only by cytokines or physical stressors, but also by neurotransmitters, such as glutamate^49,50^ and, as shown here, cholinergic agonists. Stimulation of mAChR by carbachol has previously been showed to induce activation of NF-κB in SH-SY5Ycells^51^. NF-κB plays a key role in the regulation of the inflammatory response, controlling the transcription of pro-inflammatory genes by synthesizing cytokines, including IL-1β and IL-6^52^. Therefore, the reduced levels of pNF-κB-p65 following Oxo treatment showed a good correlation with inhibitory action on IL-1β and IL-6 levels. Alternatively, since NF-κB may be activated by IL-1β^53^ or by ROS^54^, the reduction of IL-1β and ROS levels found in the present work could be responsible for pNF-κB-p65 reduction. Further experiments will be needed to examine the possible mechanism of NF-κB-p65 regulation by Oxo and to examine other cell signaling involved in the Oxo effects.

It is known that deficit of oxidants neutralization leads to an accumulation of oxidative free radicals and ROS that generate oxidative stress and cellular dysfunction^55^. In the present study we found a long lasting, up to72 h, significant reduction of global ROS levels following single Oxo injection. This result highlight that Oxo treatment may exert anti-oxidative action in the hippocampus, suggesting that it may protect cells against oxidative stress. Among ROS, we have examined the ^1^O_2_ species levels, as one of ROS with high oxidative and cytotoxic effects^56,57^. Interesting, the data obtained measuring ^1^O_2_ species levels showed a good overlap with those found analyzing global ROS levels, thus suggesting that ^1^O_2_ species generation can be one of ROS regulated by Oxo treatment. Concerning the role of mAChRs in brain responses to oxidative stress, only few and, in addition, discordant data are available. Indeed, some data reported that mAChR activation increases neuronal vulnerability to oxidative stress^13^, while others that mAChR stimulation can generate ROS, involved in neuronal-signaling pathways^14,15^. Recently, two different research groups reported that scopolamine administration induced memory deficit and increased ROS levels^58,59^, in agreement with the present data showing a significant reduction of ROS levels at all-time points following acute Oxo treatment. In contrast, here we have not found significant ROS levels change in the rat group treated with scopolamine, probably due to different timing of ROS level detection, i.e. few hours versus 72 h after scopolamine treatment.

The protection against oxidative stress needs several antioxidant enzymes such as SOD, in both its cytosolic SOD1 and mitochondrial SOD2 form, which catalyze the dismutation of superoxide anion (O_2_^−^) to oxygen and hydrogen peroxide (H_2_O_2_), thus exerting protective actions against oxidative stress^60^. In this study, we evidenced an increase of both SOD2 protein and SOD activity following Oxo treatment, and this antioxidant SOD activity appears consistent with the reduced ROS levels. Based on this result and in line with literature data showing high production of ROS in mitochondria, it is possible to make the hypothesis that mAChR activation decreases mitochondrial ROS levels through the up-regulation of SOD2. This possibility could be relevant in mitochondrial protection against injury produced by oxidative stress. Previous data showed that ACh may have neuroprotective role against oxidative stress- by enhancing the protein expressions and activity of SOD^61^, and that ACh levels are positively correlated with SOD levels^62^. Moreover, antioxidant enzyme activities, including SOD activity, are decreased in rat hippocampus following scopolamine injection^63^. Taken together, the reduction of ROS levels and the increase of SOD activity suggest a protective role of mAChRs activation against oxidative stress.

mAChRs family presents five subtypes receptors (M_1_R–M_5_R) and, among them, M_1_R, M_3_R and M_5_R are coupled to Gαq/11 family of G proteins and preferentially signal by phospholipase C activation, whereas the M_2_R and M_4_R, are coupled to Gαi/o family of G-proteins, and mainly signal by adenylyl cyclase V inhibition^1^. The subtype receptors expressed in the hippocampus are M_1_R, M_2_R and M_3_R, with high levels of M1R in neurons^64,65^ and M_3_R in glial cells^66–69^. The M_2_R and M_4_R are present in the molecular layer^8,70^ and M_5_R in CA1-CA2 Pyramidal neurons and in the subiculum^71^. In addition, M_1_R, M_2_R and M_3_R are present at both presynaptic and postsynaptic level and are involved in hippocampal synaptic plasticity^72^ contributing to the learning and memory processes^1^.

In general, activation of these mAChRs in the hippocampus regulates several functions such as synaptic plasticity, neuro-survival, cognitive responses and behaviors^6–8,18^, including stem cell proliferation and apoptosis^73,74^. Indeed, treatment with mAChR agonist carbachol sinduces proliferation of neural progenitor cells^75^, while systemic administration of pilocarpine exerts neuroprotective effects^76^. In addition, mAChRs activation may protect against apoptosis both by inducing PI3-kinase and by increasing anti-apoptotic proteins^77^ and can regulate adult hippocampal neurogenesis as well as improve behavioral deficits induced by stress, as reported by Veena *et al*.^17^. Although a specific study is needed to find out the subtype of mAChR and the related signaling involved in the observed effects of Oxo treatment, we tentatively can hypothesize the involvement of M_1_R, based on our published data showing that M_1_R mediated the Oxo-induced FGFR1 transactivation associated to neuronal axonal growth^8^.

Among glial cells, both microglia^67,68^ and astrocytes^66,69^ respond to the mAChR agonist carbachol controlling glial cytokine release, phagocytosis and movement of processes including cell migration. Interesting, carbachol activation of microglia attenuates the release activity of pro-inflammatory cytokines^78^. Accordingly, here we could find a down-regulation of pro-inflammatory cytokines levels following mAChR activation by Oxo, which could correspond to reduced release of cytokines in glial cells. However, since we did not identify the cell type involved in Oxo effects, it is not possible to discuss whether the observed effects on cytokines in basal condition could come from the stimulation of neuronal cells or glial cells.

At molecular level, both anxiety and depression are characterized by dysfunctions of multiple systems, including inflammatory mediators. Accordingly, several data support the link between the increase of inflammatory cytokines, such as IL-1β and IL-6, and the pathogenesis of mood disorders^79^. This emerging role of inflammation in the pathogenesis of depression and anxiety has recently attracted increasing interest on the immune-regulation properties of drugs showing antidepressant effects. Since in previous study we showed the ability of Oxo treatment to abolish the anxiety-like behavior induced by CRS^7^, the findings of the present work clearly showed that chronic Oxo treatment may counteract the CRS effects on IL-1β and IL-6 levels. This finding correlates with the inhibitory effect of Oxo on IL-1β and IL-6 observed following acute Oxo treatment. However, IL-1β and IL-6 levels were unchanged in the group treated for 10 days with Oxo alone, as compared to control, thus indicating that chronic Oxo treatment loses the effect observed in the acute Oxo treatment. For this result we hypothesize that mAChR receptor submitted to chronic stimulation can develop adaptation or inhibition by receptor desensitization or internalization and downregulation. In line with this hypothesis, it has been demonstrated that chronic stimulation of mAChRs induced uncoupling of the receptor from the G protein and receptor internalization or desensitization in different cell types^80,81^. Ligand-induced receptor internalization by prolonged receptor activation is relevant mechanism in regulating cellular response^82^, particularly since most drug therapies need a chronic treatment.

Numerous investigations using various animal model of anxiety-like behavior have shown pro-inflammatory effects in the brain. Variation of NF-κB, IL-1β, and IL-6 have been found in rats model anxiety-like behavior induced by food deprivation^83^ or by several stressors, including CRS, enhancing the inflammatory profile of microglia^84^. Moreover, CRS differentially regulates inflammation in mice, including IL-6^85^, and exposure to acute stress induces IL-1β protein in different brain regions in the rat^27^. Further, unpredictable chronic mild stress inducing depressive states increases pro-inflammatory cytokines levels, such a IL-1β and NF-κB in the hippocampus^86^, and maternal care deprivation increases IL-1β, IL-6 and TNF-α, whereas reduces the anti-inflammatory cytokine IL-10 level. All together these results are in line with the present data showing that CRS may induce IL-1β and IL-6. However, in this CRS model, we did not find variation of NF-κB-p65 or IL-10 and TGF-β1 protein levels.

In line with the inflammation hypothesis of anxiety-depression, anxiolytic and antidepressant effect of several drugs has been linked to a down-regulation of pro-inflammatory cytokines in the rat hippocampus^29^. For example, antidepressant agomelatine counteracts the LPS-induced up-regulation of IL-1β and IL-6 in the rat brain, and this effect seems to be mediated by inhibition of NF-κB translocation^87^. In this context, as already reported in the introduction, antidepressant effect of ketamine and β-adrenergic receptor antagonist has been associated with inhibition of pro-inflammatory responses^30,31,35^ and antidepressant fluoxetine may reduce the production of IL-1β^36^. All together these results support the present data revealing that treatment with Oxo, endowed with anxiolytic property^7^, may regulate IL-1β and IL-6.

Experimental studies have evidenced that oxidative stress is involved in the pathogenesis of anxiety and depression^38,88^. In the present study, we found increased ROS levels and reduced SOD activity in the CRS group. However, treatment with Oxo failed to counteract the increase of ROS in the CRS model, which might be likely dependent on specific molecular signals that characterize oxidative stress in the CRS model and that are out of Oxo treatment control. Further, the control group treated with chronic Oxo alone was also unable to show inhibitory effects on ROS levels, unlike the case of acute Oxo treatment. Interesting, it has been showed that chronic stress may decrease the activity of antioxidant enzymes in rat brain^89^. Accordingly, here we found that chronic Oxo treatment counteracted SOD activity reduction observed in the CRS group. However, in contrast to acute Oxo treatment, SOD activity levels were unchanged in the rat group treated with Oxo alone. Such lack of effect in the ray group treated with Oxo alone in the chronic versus acute treatment might be attributed, as already discussed above, to mAChR desensitization or internalization and downregulation after chronic treatment.

It is known that glucocorticoids, including cortisol, exert anti-inflammatory action and therefore may interfere with the observed data following treatment with Oxo. In fact, the glucocorticoid receptor, highly expressed within the rat hippocampus^90^, has been reported to influence NF-κB signaling pathway and to inhibit the expression of several cytokines genes including IL-1β itself^91^. However, in the present work, the evaluation of cortisol levels after acute Oxo treatment has shown a trend of reduction in all the examined time-points. Therefore it is possible exclude the possibility that the antioxidant effect and the reduced levels of IL-1β and IL-6 observed after acute Oxo treatment can be dependent on cortisol levels.

Concerning the CRS model we observed a trend of cortisol levels increase in all experimental groups, but in none of them it becomes significant. Therefore for pro-inflammatory cytokines restored to control levels in the CRS group after chronic Oxo treatment, we can exclude that the Oxo anti-inflammatory effect might be dependent on cortisol levels. On the other hand, pro-inflammatory cytokines were found increased in the CRS group and unchanged in the Oxo group despite the trend of cortisol levels increase.

## Conclusion

These results indicate a potential anti-inflammatory and antioxidant effect of acute Oxo treatment due to its ability to regulate pro-inflammatory cytokine and ROS levels and to increase SOD activity. Interesting, this downregulation exerted by Oxo on basal levels of IL-1β and IL-6 and on oxidative stress, reveals a muscarinic modulation of these pro-inflammatory molecules in normal condition. In the present study, we also showed that chronic Oxo treatment might exert some anti-inflammatory action in the hippocampus of rat exposed to CRS-induced anxiety-like behavior, revealing that mAChR activation, in addition to anxiolytic properties^7^, may have anti-inflammatory action. Taken together, these data suggest that mAChR agonist Oxo could potentially be used in the treatment of inflammatory component of anxiety-depression and neurodegenerative diseases, such as Alzheimer’s disease.

## Materials and Methods

### Animals care

Here we report standard information, similar to those reported in all our published papers, about the animal care and rules for the experimental use of mammals. Rats were housed in a specific pathogen-free environment, three per polypropylene cages in controlled temperature (23 ± 2 °C), humidity (50–55%) and light (12-h light/dark cycle), with access to food and water ad libitum.

The experiments were carried out in accordance with the National Institute of Health Guidelines for the Care and Use of Mammals in Neuroscience and Behavioral Research (The National Academics Press, WA, USA), with the rules and principles of the European Communities Council Directive 2010/63/EU revising Directive 86/609/EEC, in accordance with the national D.L. March 4, 2014, no. 26, and were approved by the local Animal Care Committee (OPBA) of University of Palermo, Italy and Ministry of Health, Italy. No other methods to perform the described experiments (3Rs) were found.

### Experimental design of acute Oxo treatment

Adult male Wistar rats (4 months old) were used. In the present work we used the following experimental groups, including 5 animals for each group: (1) control group treated with vehicle saline 0.9% (Ctrl); (2) acute Oxo treated group with a non-selective mAChR agonist Oxotremorine M (Oxo) (Oxotremorine 100, Sigma-Aldrich Chemical Co. St. Louise, MO, USA), receiving only one Oxo injection (0.4 mg/kg); (3) group pretreated with 1 mg/kg of mAChR antagonist scopolamine (Scopolamine hydrobromidetrihydrate, Sigma-Aldrich Chemical Co) given 20 min before Oxo (0.4 mg/kg); (4) group treated with scopolamine alone (1 mg/kg). After the scheduled treatment, control and Oxo-treated rats were sacrificed at different time points: 24, 48 and 72 h. The groups pretreated with scopolamine plus Oxo and with scopolamine alone were restricted to the time point of 72 h. The experimental schedule is shown in Fig. 1A. The Oxo dose of 0.4 mg/kg for acute treatment has been chosen in relation to our previous experience^9^. Oxo and scopolamine were dissolved in 0.9% physiological saline solution before use and the scheduled dose was given intraperitoneally (i.p.) in a volume of 0.2 ml, as well as saline treatment. At the end of the experiment, the rats were sacrificed under deep anesthesia and following perfusion with cold PBS, used to remove the blood, the hippocampi were rapidly dissected from the rat brain, quickly frozen in cooled isopentane and stored at −70 °C for later use.

Part of experimental design, above described, was used in a previous paper^9^.

### Experimental design of chronic Oxo treatment in the rat CRS model

This study was performed using the rat model of CRS previously adopted to induce anxiety-like behavior as reported in Di Liberto *et al*.^7^. Briefly, the CRS procedure was carried out once daily for 6 h and for 21 consecutive days. Rats were placed in a transparent plastic restrainer (350 ml cubage water bottle, 20 × 7 cm) with several 3 mm holes for breathing. After 21 days of CRS the experimental groups were treated for 10 days with Oxo or vehicle saline according to the below mentioned groups. Oxo at dose of 0.2 mg/kg was given i.p. in a volume of 0.5 ml, as well as vehicle saline treatment. For chronic Oxo treatment we used the dose of 0.2 mg/Kg instead of that used for acute treatment, both in relation to our previous experience^7,9^ and literature data^17,92^ and to previous preliminary test, in which the chronic treatment with the dose of 0.2 mg/Kg was better tolerated by the animals compared to 0.4 mg/kg.

The following experimental groups, with 5 rats for each group, were used: (i) control unstressed group treated with vehicle saline (Ctrl); (ii) chronic restraint-stressed group treated with saline (CRS); (iii) restraint-stressed plus Oxo-treated group (CRS + Oxo); (iiii) unstressed group treated with Oxo (Oxo). The experimental schedule is shown in Fig. 1B. After the experimental procedures, rats were sacrificed by deep anesthesia and hippocampi were removed from brain for the detection of inflammatory and oxidant marker levels.

### Western blotting

Western blotting was performed as previously described by Frinchi *et al*.^93^. Dissected hippocampal tissue was homogenized in cold radioimmunoprecipitation assay (RIPA) buffer (50 mM Tris, pH 7.4, 150 mM NaCl, 1% Triton, SDS 0.1%), supplemented with protease inhibitor cocktail (Sigma-Aldrich P8340) and phosphatase inhibitor cocktail (Sigma-Aldrich, P5726). Samples were sonicated (30 pulsations/min), quantified by the Lowry method^94^ and stored at −80 °C. Protein Samples (30–50 μg per lane) and molecular weight marker (161-0376 BIO-RAD) were run on 10% polyacrylamide gel and electrophoretically transferred onto nitrocellulose membrane (RPN303E, Hybond-C-extra, GE Healthcare Europe GmbH). The membranes were incubated for 1 h in blocking buffer (1x TBS, 0.1% Tween-20, 5% w/v nonfat dry milk) and incubated with gentle shaking overnight at −4 °C with specific antibody diluted in blocking buffer. For detection of NF-κB, phosphorylated NF-κB (pNF-κB), SOD1 and SOD2, as already described in Mudò *et al*.^95^, the following antibodies and procedures were used: rabbit polyclonal anti-NF-κB-p65 subunit 1:500 (SC-372 Santa Cruz Biotechnology);mouse-monoclonal anti-pNF-κB-p65 1:500 (Sc-136548 Santa Cruz Biotechnology); rabbit polyclonal anti-SOD1 1:1000 (Sc-11407 Santa Cruz Biotechnology); mouse anti-SOD2 1:500 (SOD2; sc-137254, Santa Cruz Biotechnology). The day after, membranes were washed three times for 10 min with TBS/T and incubated for 1 h at room temperature with goat anti-Rabbit IgG (sc-2004 Santa Cruz Biotechnology) or goat anti-mouse IgG (Sc.7076 Cell Signaling Technology) horseradish peroxidase-conjugated diluted 1:10000. After three washings with TBS-T, immunocomplexes were visualized with chemiluminescence reagent (RPN2236, GE Healthcare Europe GmbH) according to the manufacturer’s instructions. The Hyperfilm (ECL-films 28906837, GE Healthcare Europe GmbH) were developed using Kodak developer and fixer (catalog no. 1900984 and 1902485, Kodak GBX, Eastman Kodak). For the normalization of quantitative evaluation of bands each membrane was stripped at 65 °C for 30 min in buffer containing NaCl 137 mM, TrisHCl 20 mM pH 7.6 and β-mercaptoethanol 0.01%. After two washings with TBST, the membranes were reprobed with anti-β-Actin antibody (sc-47778, Santa Cruz Biotechnology). The densitometric evaluation of bands was performed by measuring the optical density (O.D.) using the Image J software (Rasband WS, ImageJ, U.S. National Institutes of Health, Bethesda, Maryland, USA, http://rsb.info.nih.gov/ij/, 1997–2018). All uncropped images are presented in Supplementary Figs S1–S4.

### Measurement of pro-inflammatory or anti-inflammatory cytokines by ELISA-assay

Concentrations of IL-1β, IL-6, IL-10 and TGF-β1 were measured in the hippocampus homogenates (20 mg of tissue sample) using enzyme-linked immunosorbent assay **(**ELISA) kits for rat (Cloud-Clone Corp, Wuhan, Hubei) according to the manufacturers’ protocols and as reported in Zizzo *et al*.^96^ and in Mudò *et al*.^95^.

### ROS analysis

The procedures described here are found in previous papers^95,97^, in order to asses ROS generation we used two fluorescent probes: (1) dichlorofluorescein diacetate (DCFH-DA); (2) Singlet Oxygen Sensor Green (SOSG). (1). The nonselective DCFH-DA probe was used to asses several ROS generation, mainly including hydrogen peroxide (H_2_O_2_), hydroxyl radical (^*^OH) and peroxyl radical (ROO*), a lipid peroxide associated to the cellular membrane. Therefore, we used the DCFH-DA assay as adequate procedure to measure the global variation of ROS levels and reveal cell oxidative stress^98^. Tissue from rat hippocampus (10 mg) were suspended on 1000 µl of PBS1X with 10 μl of protease inhibitors (Amersham Life Science, Munich, Germany). Then, samples were incubated with 1 mM dichlorofluorescein diacetate (DCFH-DA) for 10 min at room temperature in the dark. The conversion of non-fluorescent DCFH-DA to the highly fluorescent compound 20,70-dichlorofluorescein (DCF) by esterase activity was used to monitor the presence of peroxides due to the oxidative burst in the brains. Therefore, the emitted fluorescence is directly proportional to the concentration of ROS. The samples were analyzed by fluorimeter (Microplate reader WallacVictor 2–1420 MultilabelCounter; PerkinElmer, Inc.), using the excitation filter set at 485 nm and the emission filter set at 530 nm. Relative ROS production was expressed as the change in fluorescence of experimental groups compared with that of the appropriate controls (100%). (2) The SOSG probe, which is considered to be highly selective and sensitive singlet oxygen (^1^O_2_) indicator^99,100^, although some criticism about sensitivity^101^, was used to selectively evaluate the ^1^O_2_ species generation. We have chosen to evaluate the ^1^O_2_ species generation as one of ROS with high oxidative and cytotoxic effects^56,57^. To valuated the level of ^1^O_2_, 10 mg of tissue from rat’s hippocampus was weighed and homogenate on 1000 μl of PBS1X. After homogenizing the sample tubes were centrifuged at 10,000 g at 2 °C for 5 min. 48 μL of supernatant of each sample was mixed with 50 μL of the reagent buffer and 2 μL of 5 mM of SOSG agent (Molecular Probes, USA). 80 μL of each sample mixture was added into a well of 96-well plate (Black Microtiter Plate, Thermo Scientific, Finland) and covered with transparent lid. The fluorescence signal was measured at excitation 488 nm and emission 525 nm by a spectrophotometer GloMax® Discover System (Promega, USA).

### SOD activity levels

The procedure described here is reported in Mudò *et al*.^95^. The Hippocampus of rats was homogenized in PBS with protease inhibitors (Amersham Life Science, Munich, Germany). To remove insoluble material, tissue lysates were sonicated and centrifuged (14.000 rpm, at 4 °C, for 30 min). In the supernatant, total proteins were quantified by Bio-Rad Bradford Protein Assays. Volume corresponding to 50 μg of protein were used for total SOD enzymatic activity measurement, by using the SOD assay kit (Sigma-Aldrich) according to manufacturer’s instructions. Absorbance was measured by using the iMark™ Microplate Absorbance Reader at 450 nm.

### Cortisol levels

The procedure described here is reported in Mudò *et al*.^95^. Rats of all the experimental groups were decapitated under deep anesthesia between 11:00–12:00 am and blood was collected in tubes coated with EDTA. Blood samples were centrifuged at 4,000 × *g* at 4 °C for 10 min and the supernatant was stored at −80 °C. The plasma levels of cortisol were measured using automated electrochemiluminescence immunoassay (Roche Diagnostics Elecsys Cortisol II assays and COBAS E801) and values were expressed in ng/ml. The minimum level of detection for assays of cortisol was 0.15 ng/ml.

### Statistical analysis

The statistical analysis procedure used was described in Mudò *et al*.^95^. The results are presented as mean ± SE, and in some case are expressed as arbitrary units, with controls equal to 1, or as percentage of control. Statistical evaluation was performed by one-way ANOVA, followed by Fisher’s Protected Least Significant Difference (PLSD) test. Two-way analysis of variance (ANOVA) was performed to assess the main effect of CRS (stress) and Oxo treatment (treatment), and the interaction (stress × treatment). When ANOVA was found significant, intra- and inter-group differences were measured by student *t-*test. Differences in P-value less than 0.05 were considered statistically significant.

## Supplementary information

Supplementary information
