# Supplementary material for: Anti-inflammatory and antioxidant effects of muscarinic acetylcholine receptor (mAChR) activation in the rat hippocampus
Source: Sci Rep. 2019 Oct 2;9:14233. doi: 10.1038/s41598-019-50708-w (PMC6775129; doi:10.1038/s41598-019-50708-w)
Supplement: Supplementary file 1 — Supplementary information [file 41598_2019_50708_MOESM1_ESM.pdf]

Anti-inflammatory and antioxidant effects of muscarinic acetylcholine receptor (mAChR) activation in the rat hippocampus.

Frinchi Monica[1]$, Nuzzo Domenico[2]$, Scaduto Pietro[1], Di Carlo Marta[2], Massenti Maria F.[3], Belluardo Natale[1]§, Mudò Giuseppa[1*]§.

[1]Department of Experimental Biomedicine and Clinical Neurosciences, div. of Human Physiology, University of Palermo, 90134 Palermo, Italy

[2]Institute of Biomedicine and Molecular Immunology "Alberto Monroy" (IBIM), Consiglio Nazionale delle Ricerche (CNR), 90146 Palermo, Italy.

[3]Department of Sciences for Health Promotion and Mother and Child Care "Giuseppe D'Alessandro", University of Palermo, 90134, Palermo, Italy

$Co-first authors

§Co-senior authors,

*Corresponding author: Department of Experimental Biomedicine and Clinical Neurosciences, div. of Human Physiology, University of Palermo, Corso Tukory 129, 90134 Palermo, Italy. E-mail giuseppa.mudo@unipa.it

S1

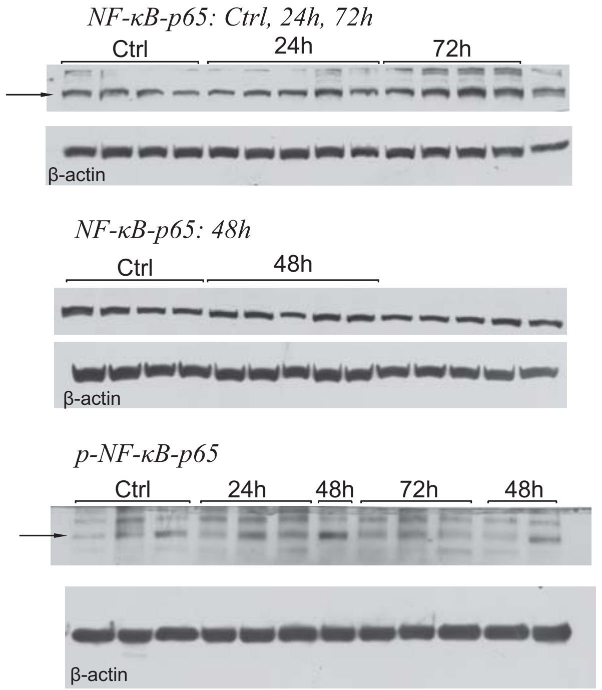

Uncropped bands for data shown in fig 3

S2

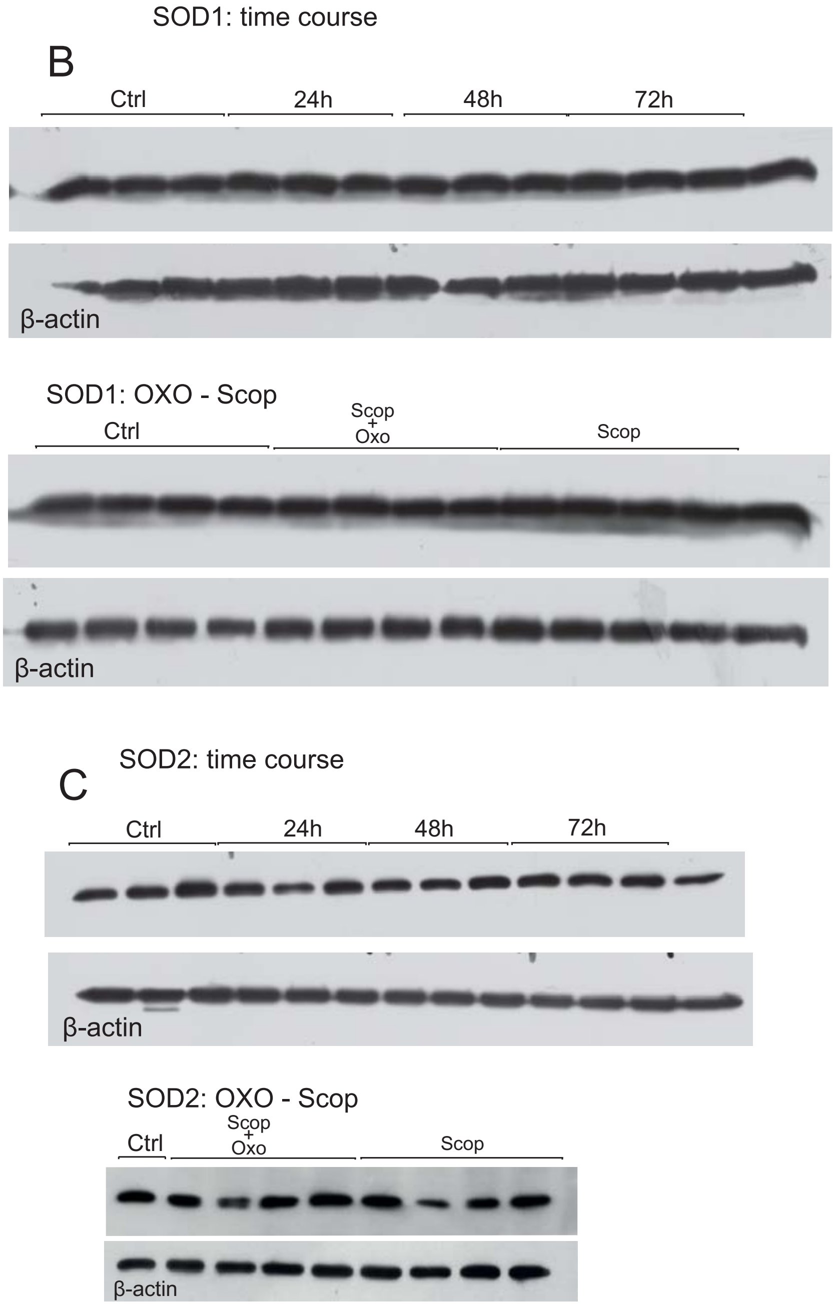

Uncropped bands for data shown in fig 4

S3

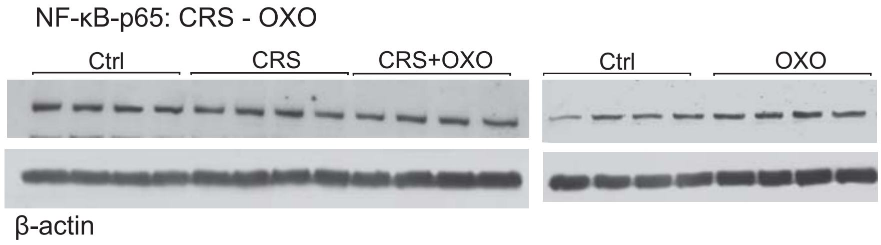

Uncropped bands for data shown in fig 5

S4

B

SOD1: CRS - OXO

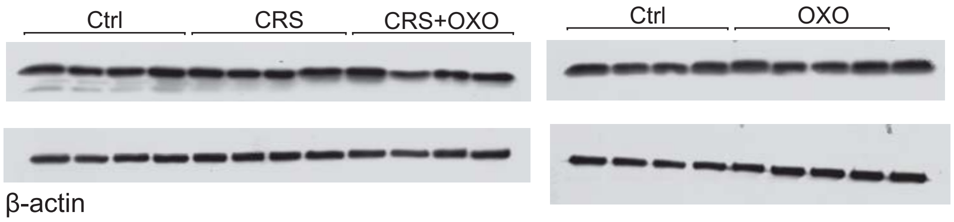

C

SOD2: CRS - OXO

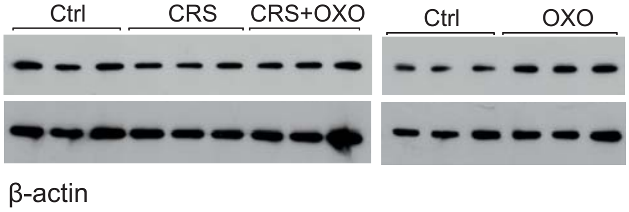

Uncropped bands for data shown in fig 6
